# Hierarchical Co-based Porous Layered Double Hydroxide Arrays Derived via Alkali Etching for High-performance Supercapacitors

**DOI:** 10.1038/srep13082

**Published:** 2015-08-17

**Authors:** Nasser Abushrenta, Xiaochao Wu, Junnan Wang, Junfeng Liu, Xiaoming Sun

**Affiliations:** 1 State Key Laboratory of Chemical Resource Engineering, Beijing University of Chemical Technology, Beijing 100029, China

## Abstract

Hierarchical nanoarchitecture and porous structure can both provide advantages for improving the electrochemical performance in energy storage electrodes. Here we report a novel strategy to synthesize new electrode materials, hierarchical Co-based porous layered double hydroxide (PLDH) arrays derived via alkali etching from Co(OH)_2_@CoAl LDH nanoarrays. This structure not only has the benefits of hierarchical nanoarrays including short ion diffusion path and good charge transport, but also possesses a large contact surface area owing to its porous structure which lead to a high specific capacitance (23.75 F cm^−2^ or 1734 F g^−1^ at 5 mA cm^−2^) and excellent cycling performance (over 85% after 5000 cycles). The enhanced electrode material is a promising candidate for supercapacitors in future application.

The depletion of fossil fuels and the global warming crisis have led the international community to develop alternative and clean energy system. Batteries and fuel cell technologies are being heavily researched to meet this energy demand^1^. Fuel cells and batteries usually possess higher energy density, but both technologies lack the high power density which can be supplied by fuel^2^,^3^,^4^. Currently, lithium ion batteries are dominantly powering most of today’s portable electronics due to the high efficiency and energy density, but the high expense and possible unsafety originated from the flammability of organic electrolytes and high reactivity of Li containing materials are two major concerns for our daily life^5^,^6^. Supercapacitors (SCs) possess numerous advantages that complement many deficiencies of other energy storage devices. Their long cycling lifetime, high power density, and low maintenance cost have aroused great interest academically and commercially, making them more attractive and versatile as high-powered energy storages. They are capable of bridging the gap between batteries/fuel cells with low power densities and energy-lacking capacitors^7^. Pseudocapacitor devices based on faradic storage behavior have recently attracted vast attention because they are able to offer higher capacitance and energy density compared with EDLC^8^. As one of the most wonderful pseudocapacitive materials, ruthenium oxide (RuO_2_) has exhibited excellent high specific capacitance, good electrical conductivity, and high chemical stability. However, the high cost and rareness of the Ru element limit its application in supercapacitors^9^,^10^,^11^. Therefore, low cost transition metal oxides or hydroxides with variable valence, such as NiO^12^, Co_3_O_4_^13^,^14^,^15^, MnO_2_^16^, Ni(OH)_2_^17^, and Co(OH)_2_^18^ were investigated extensively to substitute noble metal as the electrode materials for supercapacitor. Especially, nano-size metal material not only store energy like electrostatic carbon materials but also exhibit electrochemical faradaic reactions between electrode materials and ions within appropriate potential windows^19^,^20^. Thus, they often have theoretical specific capacity values larger than 2000 F g^−1^ (e.g. 3560 F g^−1^ for Co_3_O_4_^13^ and 2573 F g^−1^ for NiO^14^) due to their multiple oxidation states that result in a rich variety of redox reactions for pseudo capacitance generation^21^.

Layered double hydroxides (LDHs), have attracted increasing interest from both academic and industrial angles due to their wide applications in various areas^22^,^23^,^24^. Recently, LDHs materials, containing transition metal, have been reported to act as promising electrode materials for ECs because of their relatively low cost, high redox activity, and environmentally friendly nature^25^. LDHs with vertically aligned structures have exhibited great potential in SCs^26^,^27^,^28^,^29^. To further improve the performance of 2D structure, creating holes in the nanosheets to increase the porosity is regarded as an effective method. In general, a porous structure with a large surface area significantly improves the charge transfer and capacitance of electrode^30^. Our group has fabricated a thin mesoporous cobalt carbonate hydroxide (MPCCH) nanosheet array by selectively etching the Al element of a CoAl LDH thin film in highly concentrated NaOH solution^31^. The specific capacitance of the MPCCH could reach 1,075 F g^−1^ at 5 mA cm^−2^, which was much higher than that of the CoAl LDH precursor. On the other hand, constructing hierarchical nanoarray architecture has been demonstrated as one of the most efficient routes to improve the electrochemical energy storage performance of the electrode materials^2^. Specifically, hierarchical nanoarray architecture can offer a direct growth of the active materials on conductive substrates, which ensures a good electric contact and consequently enhances the rate capability. Moreover, the three dimensional structure possesses a much higher contact area with the electrolyte and thus offers a larger electrochemical surface area. In addition, the hierarchical nanoarray may even circumvent the conflict between the mass loading of electrochemically active material and its utilization efficiency^32^,^33^,^34^,^35^,^36^.

Herein, we successfully synthesized hierarchical Co-based LDH nanoarrays (Co(OH)_2_@CoAl LDH) by a facile two-step hydrothermal reaction. By immersing the precursor in a highly concentrated alkaline solution, we obtained various hierarchical porous LDH (Co(OH)_2_@PLDH) nanoarrays. The synthesis process of the material is schematically shown in Fig. 1. Firstly, Co(OH)_2_ nanosheet (NS) arrays were synthesized on the 3D macroporous structured Ni foam via hydrothermal method. Subsequently, by co-precipitation of Co^2+^ and Al^3+^ under the similar conditions, CoAl LDH nanosheet arrays were grown on Co(OH)_2_ NS array. The final product, Co(OH)_2_@PLDH, was derived through alkaline etching in concentrated NaOH solution, which was aimed to remove the Al to construct a mesoporous structure. The hierarchical nanoarchitecture as well as porous structure benefit the material in electrochemical performance. As an advanced electrode for SC, this integrated electrode showed both higher areal capacitance and specific capacitance than those of Co(OH)_2_ NS arrays and Co(OH)_2_@CoAl-LDH nanoarrays. Even after 5000 cycles of charge and discharge, no significant decrease in capacitance was observed. Additionally, the effect of etching time to enhanced supercapacitive performance was reported.

## Results

### Structure and morphology

**Figure 2:**
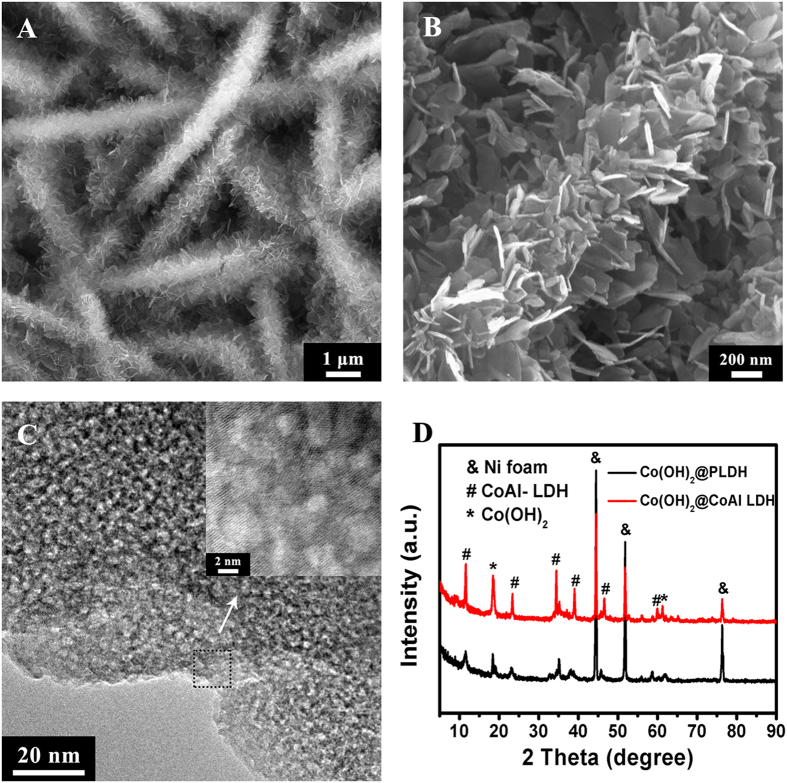
Low (**A**), high (**B**) magnification SEM and (**C**) HRTEM images of hierarchical Co(OH)_2_@PLDH arrays; (**D**) XRD patterns of Co(OH)_2_@PLDH and hierarchical Co(OH)_2_@CoAl LDH arrays.

The construction of hierarchical Co(OH)_2_@PLDH arrays derived from alkali etching is fully demonstrated in the SEM images at different magnification (Fig. 2). For comparison, the samples after the first and second hydrothermal process, defined as Co(OH)_2_ NS and Co(OH)_2_@CoAl LDH respectively, were shown in Figure S1 and S2. At first, Co(OH)_2_ NS with size of 4 μm was grown on the surface of Ni foam (Figure S2 a,b). After second hydrothermal process, the smaller-sized LDH nanosheets of 200 nm were vertically grown outside of the Co(OH)_2_ NSs (Figure S2 c,d). Devised via alkaline etching, Co(OH)_2_@PLDH arrays was obtained. The low magnification SEM image of Co(OH)_2_@PLDH arrays revealed that the substrate surface was uniformly covered by well-aligned nanosheet arrays with an average length about 4 μm (Fig. 2A) and the high magnification SEM image in Fig. 2B illustrate that the secondary porous nanosheets LDH were estimated to around 200 nm, which formed highly dense film around the Co(OH)_2_ NSs. The HRTEM image in Fig. 2C exhibited that the secondary LDH nanosheets possessed porous structure and crystallized characteristic, in which two obvious lattice spaces of (006) and (012) planes for LDH structure were observed.

The extraction of Al from the secondary LDH precursor did not affect the phase change dramatically, which was explained in the XRD pattern in Fig. 2D. XRD data of Co(OH)_2_@PLDH and Co(OH)_2_@CoAl LDH showed a series of similar Bragg reflections (XRD pattern for Co(OH)_2_ NS was also shown in Figure S1 for comparison). In XRD pattern of Co(OH)_2_@CoAl LDH, the peaks marked ‘#’ were in good agreement with the well-known LDH Co_6_Al_2_CO_3_(OH)_16_•H_2_O (JCPDF: 51-0045) with the characteristic peaks of (003) and (006) planes, which revealed an interlayer distance of 0.74 nm, indicating the occupying of CO_3_^2−^ ions and water molecules in the interlayer spaces^37^. While the peaks marked ‘*’ referred to the primary Co(OH)_2_ (JCPDF: 45-0031), and no reflections due to crystalline impurities was observed (the peaks marked ‘&’ donate the Ni substrate). The XRD pattern for Co(OH)_2_@PLDH still exhibited Bragg reflections at the same 2θ values, indicating that the layer structure retained. However, the overall intensities of XRD pattern decreased while the relative of intensity of peaks were unchanged. The peak widths at half maximum increased, indicative of a smaller size of nanosheet and decreased crystallinity^31^.

**Figure 3:**
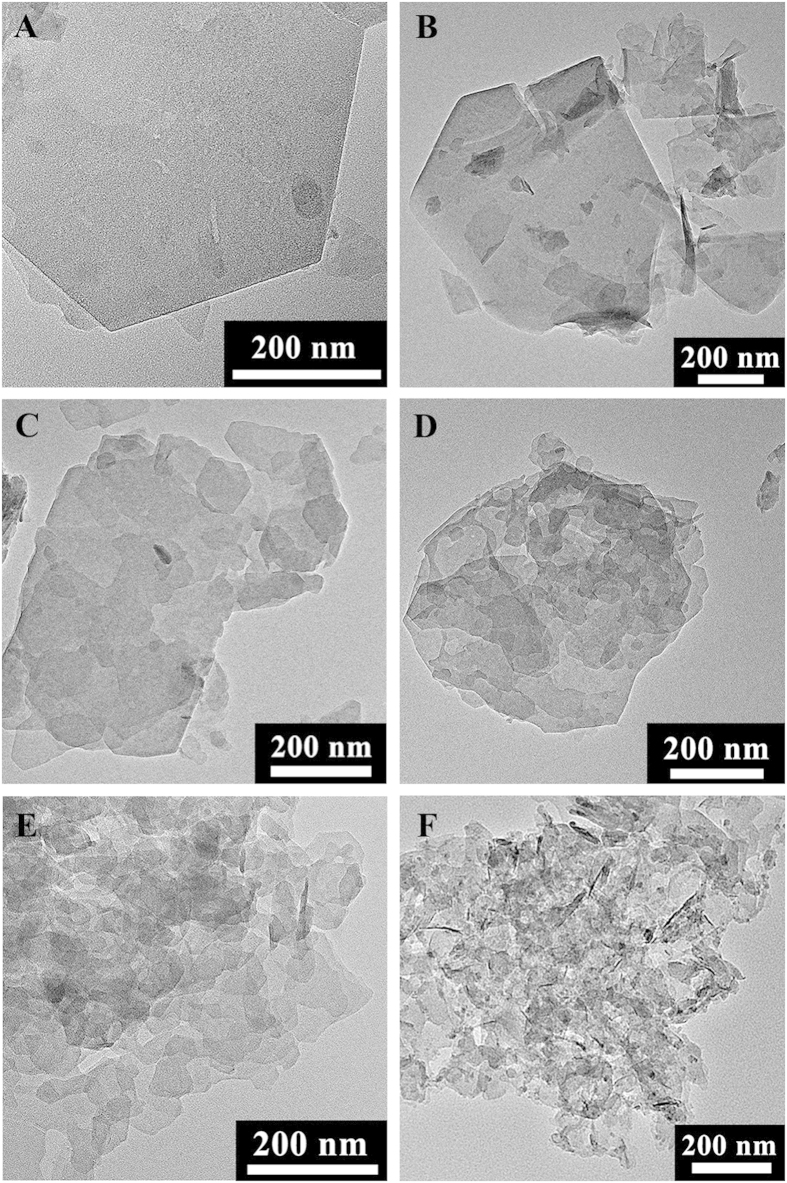
The TEM images of various samples with different alkali etching time (**A**) Co(OH)_2_@CoAl LDH; (**B**) Co(OH)_2_@PLDH-6; (**C**) Co(OH)_2_@PLDH-12; (**D**) Co(OH)_2_@PLDH-18; (**E**) Co(OH)_2_@PLDH-24; (**F**) Co(OH)_2_@PLDH-48.

The morphology evolution of Co(OH)_2_@PLDH at different alkali etching time were investigated by TEM and EDS. The resultant samples were denoted as Co(OH)_2_@PLDH-X (‘X’ is the alkali etching time in hours, X = 6, 12, 18, 24 and 48). Figure (3A–F) showed the TEM images of the secondary structure of Co(OH)_2_@CoAl LDH and various Co(OH)_2_@PLDH-X. Before soaking the hierarchical Co(OH)_2_@CoAl LDH nanosheet arrays in high concentrated basic solution, the surface of hexagonal nanosheet is relatively smooth. Further prolonging the soaking time would result in much rougher and extensive cracking. Small holes with several to tens of nanometers in size were clearly observed in cracked nanosheets, which is because that the easy dissolution of Al^3+^ in strong base would create defects in the pristine LDH lattice. Compared with the prodromal Co(OH)_2_@CoAl LDH, the alkali treated samples were expected to have a much higher specific area. The Co: Al atomic ratio of modified films at different etching time were determined by EDS (Figure S3) and detailed data was listed in Table S1, which indicates a tendency of increase for the Co: Al atomic ratio with the etching time growing. Combining the EDS, SEM, and TEM data, it was concluded that alkali etching in the concentrated basic solution led to the removal of Al cations from CoAl LDH and formation of holes and cracks in nanosheets gradually. The process thinned the sheets, increased the surface area and facilitated the penetration of electrolytes into electrodes.

### Electrochemical analysis

**Figure 4:**
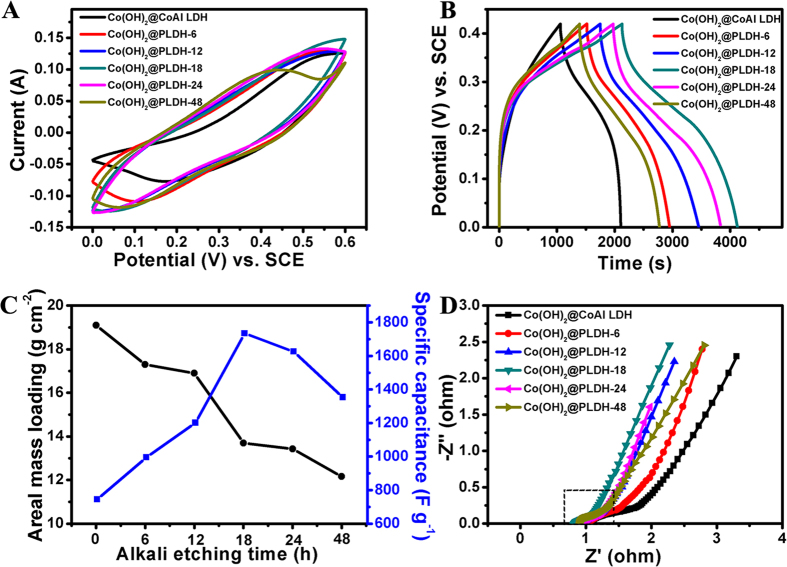
Electrochemical characterization of the Co(OH)_2_@PLDH-X at different alkali etching time. (**A**) CV curves at 10 mV s^−1^ ; (**B**) galvanostatic charge/discharge curves at 5 mA cm^−2^; (**C**) areal mass loading per square centimeter and specific capacitance; (**D**) Nyquist plots of EIS.

To assess their potential for supercapacitor application, the electrochemical performance of hierarchical Co(OH)_2_@PLDH arrays derived via alkali etching were investigated by cyclic voltammograme and galvanostatic charge/discharge measurements. Fig. 4A showed the typical CV curves of Co(OH)_2_@CoAl LDH and Co(OH)_2_@PLDH-X that immersed in NaOH for different times at a scan rate of 10 mV s^−1^ within a potential range of 0 ~ 0.6 V (vs. SCE). For each CV curves, the set of redox peaks observed indicated the existence of a faradic process which was ascribed to interconversion of Co(II)/Co(III) couple^38^. The sample with 18 hours of alkali etching, denoted as Co(OH)_2_@PLDH-18, show the best capacitive behavior among various samples, which was also proved by the galvanostatic charge/discharge curves at a current density of 5 mA cm^−2^ in Fig. 4B. With the alkali etching time rising, the areal mass loading per square centimeter decrease gradually, while the specific capacitance calculated from Equation 1 present a trend of increase, which was shown in Fig. 4C. Therefore, we believe that alkali etching treatment of the electrodes enhanced the contact area between the electrode and electrolyte, resulting in more efficient utilization of the active material. However, excessive corrosion might lead to the structure destroy and arrays’ collapse. That is the reason that there was an obvious peak value in the curve of specific capacitance, which illustrated that Co(OH)_2_@PLDH-18 was the best performing sample. The electrochemical surface area (ESA) can be evaluated the roughness of the electrode surface^39^, so the electric double layer capacitance (EDLC) was tested to show the ESA change in different etching time. Based on the CV curves at different scan rate in the region of 0.25 ~ 0.3 V, in which no apparent faradaic process occur, the EDLC of different samples could be calculated in Figure S4^40^. With longer etching time in basic solution, the electrode showed larger EDLC, which helps to explain the best performance of Co(OH)_2_@PLDH-18. Although Co(OH)_2_@PLDH-24 and Co(OH)_2_@PLDH-48 possessed a bit of larger EDLC, they may not maintain the good contact to primary structure and substrate as Co(OH)_2_@PLDH-18. The mass loadings, ESA, specific capacitance and areal capacitance were listed in Table 1, which illustrated the detailed data of the electrochemical performances for various samples. Electrochemical impendence spectroscopy (EIS) was also performed with an open circuit potential to investigate the electrode kinetics in these electrode samples (Fig. 4D). From the intersection point with real axis in high frequency range, samples with different alkali etching time showed similar ohmic resistance values (Figure S5 showed the details within the dotted line of Fig. 4D), indicating that the internal resistance experienced a slight change. On the other hand, the low-frequency tails for these samples were totally different. The slope of the curves showed the Warburg impedance which represents the electrolyte diffusion in the porous electrode and proton diffusion in host materials^41^. With the increasing atomic ratio of Co and Al, the sample with longer-time alkali etching showed more vertical line in this figure, suggesting that it would have lower diffusion resistance if it contained less aluminum in the sample. Al is not a promising element electrochemical storage owing to its poor redox reaction, but it has been reported^42^ that Al doping could help transition metal materials exhibit better electrochemical storage and higher capacity.

**Figure 5:**
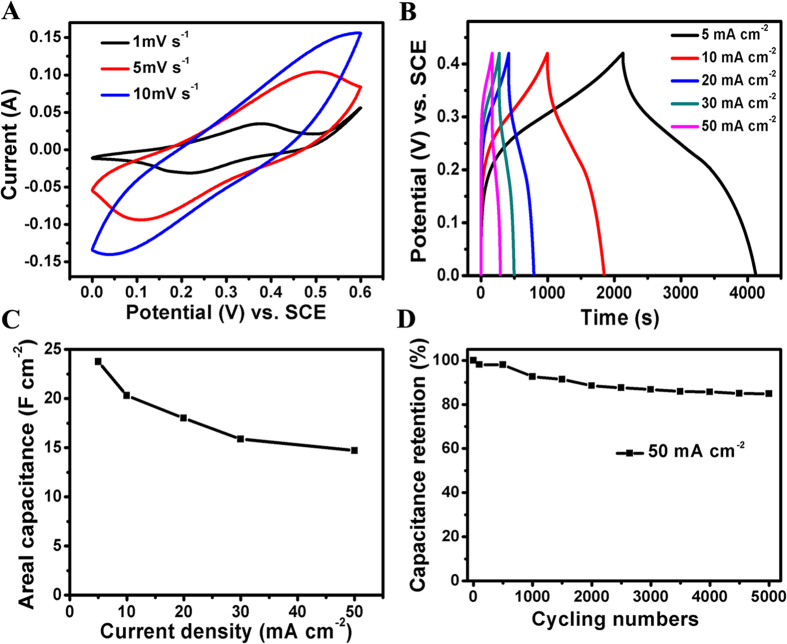
Electrochemical characterization of the hierarchical Co(OH)_2_@PLDH-18 array electrode. (**A**) CV curves at 1 ~ 10 mV s^−1^; (**B**) and (**C**) galvanostatic charge/discharge curves and areal capacitance values at different current densities; (**D**) cycling stability at 50 mA cm^−2^.

To demonstrate the advantages of the best electrode (Co(OH)_2_@PLDH-18) originating from its unique structure, CV curves at different scan rates were given in Fig. 5A. The pair obvious redox peaks was attributed to the redox couples of Co(II)/Co(III), as previous research on Co-based hydroxide had indicated^36^. The CV with a potential range of 0 ~ 0.6 V (vs. SCE) manifested that with the increase of the scan rate, the redox current increased and the anodic peak and cathodic peaks shifted toward positive and negative potential, respectively. The location of the redox peaks showed a slight shift with Co(OH)_2_@CoAl LDH, and it was due to the different environment of cobalt, which was proved in XPS pattern shown in Figure S6. Figure 5B gave the results for Co(OH)_2_@PLDH-18 electrode over the potential range of 0 ~ 0.42 V at various current rates. The discharge curves showed a significant deviation from straight and flat lines, indicating that the capacitance mainly comes from the faradic redox reactions, which is consistent with the CV curves. Calculated from the discharge curves (Fig. 5C), the areal capacitance delivered as high 23.75 F cm^−2^ at a current density of 5 mA cm^−2^, while the capacitance could be retain at 14.7 F cm^−2^ as the current density increased to 50 mA cm^−2^, showing a rate capability of ~62%. When it comes to the specific capacitance, the value was still acceptable (1734 F g^−1^ at 5 mA cm^−2^) .The remarkable rate capability as well as the ultrahigh areal capacitance manifested the effectiveness of constructing hierarchical and porous architecture. For comparison purposes, the Co(OH)_2_ NS and Co(OH)_2_@CoAl LDH were evaluated using the same measurements (Figure S7). It was found that both Co(OH)_2_ NS and Co(OH)_2_@CoAl LDH showed much inferior performance, which indicated that Co(OH)_2_@PLDH-18 has the advantages on both hierarchical nanoarchitecture and porous structure. To verify their benefit, nitrogen sorption isotherm and pore size distribution were characterized for Co(OH)_2_ NS, Co(OH)_2_@CoAl LDH and Co(OH)_2_@PLDH-18. The result showed a type-IV isotherm (Figure S8), which indicated the mesoporous characteristic of these samples. From Table S2, it illustrated that Co(OH)_2_@PLDH-18 possessed larger specific surface area than its precursor.

As long-time cycling stability is also an important factor in supercapacitors, cycle charge discharge testing for Co(OH)_2_@PLDH-18 electrode was employed to examine the service life at a current density of 50 mA cm^−2^ (Fig. 5D). The capacity almost remained stable with a minor fluctuation over the initial 500 cycles and subsequently decreased gradually. After 5000 cycles, Co(OH)_2_@PLDH-18 electrode showed an overall decay of ~15% in final status.

In summary, a simple, low-cost, template-free and environment-friendly method has been developed to fabricate a hierarchical Co-based porous layered double hydroxide arrays by immersing a Co(OH)_2_@CoAl LDH precursor in highly concentrated alkaline solution. Such a novel strategy combined the superiorities of hierarchical nanoarchitecture and porous structure, which was beneficial for the electrochemical storage performance. The optimized Co(OH)_2_@PLDH-18 electrode showed a high areal as well as specific capacitance (23.75 F cm^−2^ or 1734 F g^−1^) at 5 mA cm^−2^. Besides, the rate retention and cycling stability was ~62% and 85% respectively, which illustrated its excellent property. This advanced supercapacitor electrode is attributed to its outstanding morphology, meanwhile appropriate porous structure and Al-doping. The fabrication approach via alkali etching described in this work should be applicable for other amphoteric metal materials, and it could also be applied for constructing promising electrodes in electrochemical storage systems.

## Methods

The chemicals involved in this experiment were all analytically pure and none of them were treated with a second purification. All the following structure was based on a piece of nickel foam (about 2 cm × 3 cm). The foam was cleaned by HCl solution for less than 3 minutes to remove the inactive oxide layer, then it was washed with deionized water as well as ethanol for 5 minutes each. After being dried, the sample was weighted before the reaction.

### Synthesis of Co(OH)_2_ NS

In a typical experiment, one foam was put to recline the wall of Teflon-lined stainless-steel autoclave, which contained homogeneous solution of Co(NO_3_)_2_•6 H_2_O (2 mmol), NH_4_F (8 mmol), CO(NH_2_)_2_ (10 mmol) and 36 ml deionized water. Afterwards, the autoclave was sealed and maintained at 100 °C for 6 hours. It was cooled down spontaneously after the reaction. Cleaned with deionized water and ethanol, the resulted intermediate product was the primary structure on nickel foam, which was labeled as Co(OH)_2_ nanosheet (NS).

### Synthesis of Co(OH)_2_@CoAl LDH

The secondary structure CoAl LDH nanosheet array was synthesized upon the primary structure via a facile hydrothermal approach. Similar to the first step, the intermediate product Co(OH)_2_ NS on nickel foam was set in the Teflon-lined stainless-steel autoclave as a slope. The uniform solution in the autoclave included Co(NO_3_)_2_•6 H_2_O (2 mmol), Al(NO_3_)_3_•6 H_2_O (2 mmol), NH_4_F (8 mmol), CO(NH_2_)_2_ (10 mmol) and 36 ml deionized water. Subsequently, the autoclave was kept in 100 °C for 24 hours. When cooled down, the sample was cleaned with deionized water and ethanol. The secondary structure was completed and we defined this hierarchical product as Co(OH)_2_@CoAl LDH.

### Synthesis of Co(OH)_2_@PLDH

The final step was to treat Co(OH)_2_@CoAl LDH with alkali for etching the structure (eliminate Al) and improve the surface area, which displayed a porous morphology. All the pink samples were divided into 5 groups to be immersed in 5 mol•L^−1^ NaOH solution for 6, 12, 18, 24, 48 hours, respectively, at room temperature. The corresponding products were defined as Co(OH)_2_@PLDH-X (X = 6, 12, 18, 24, 48). Then came the cleaning procedure with deionized water and ethanol. The weight of final dried products were acquired accurately and the specific mass loading statistics were achieved by subtracting unit area weight of product with that of initial nickel foam.

### Characterization

X-ray powder diffraction (XRD) patterns were documented on an X-ray diffractometer (Rigaku D/max 2500) with a scan rate of 10°/min ranging from 5° to 90°. The morphology and size of the samples were investigated with a TEM system (FEI) operating at 200 kV and a field-emission SEM (Zeiss SUPRA 55) working at 20 kV, respectively. Moreover, the energy dispersive spectrometer (EDS) was used to determine the composition of samples separated from substrate. X-ray photoelectron spectroscopy (XPS) was carried out by using a model of ESCALAB 250.

### Electrochemical Measurements

The electrochemical performance was measured by electrochemical workstation (CHI 660D, Chenghua, Shanghai). The testing system was under room temperature condition and contained a three-electrode glass cell. Fresh film of the product (1 cm × 1 cm) was used as the working electrode. The counter electrode was a platinum electrode while the reference electrode was a saturated calomel electrode. The electrolyte was 2 mol•L^−1^ KOH aqueous solution.

### Electrochemical Performance Calculation

The specific capacitance *C* was calculated by galvanostatic discharge curves with Equation 1. In the equation, the symbol “*I*” stood for current, “*ΔV*/*Δt*” was the slope of discharge curve, and “*m*” was the mass of active materials on the electrode. When replacing the “*m*” with unit area, the derived equation could serve as the approach to calculate areal capacitance.


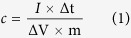


## Additional Information

**How to cite this article**: Abushrenta, N. *et al.* Hierarchical Co-based Porous Layered Double Hydroxide Arrays Derived via Alkali Etching for High-performance Supercapacitors. *Sci. Rep.* **5**, 13082; doi: 10.1038/srep13082 (2015).

## Supplementary Material

Supplementary Information

## Figures and Tables

**Figure 1:**
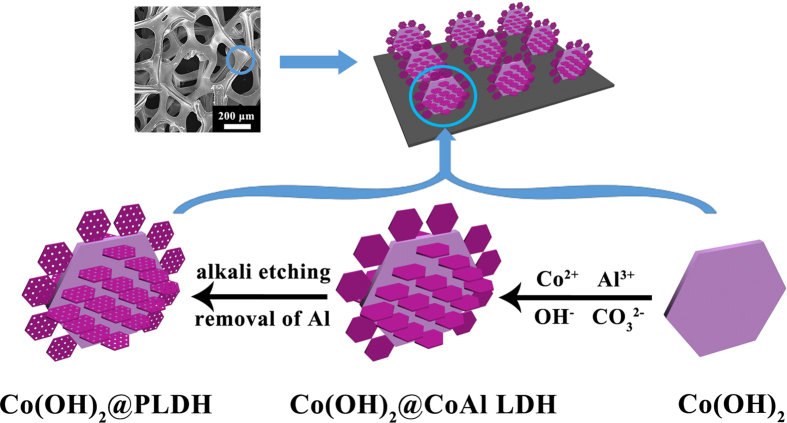
Schematic illustration for the fabrication process of hierarchical Co(OH)_2_@PLDH arrays on Ni foam.

**Table 1:** Electrochemical results of the various samples with different alkali etching time.

**Samples**	**Massloading(mg cm**^−**2**^)	**Electrochemicalsurface area (F cm**^−**2**^)	**Arealcapacitance(F cm**^−**2**^)	**Specificcapacitance(F g**^−**1**^)
Co(OH)_2_@CoAl LDH	19.1	1.34	13.44	744.8
Co(OH)_2_@PLDH-6	17.3	1.64	17.21	995.3
Co(OH)_2_@PLDH-12	16.9	1.91	20.30	1201.4
Co(OH)_2_@PLDH-18	13.7	2.33	23.75	1734
Co(OH)_2_@PLDH-24	13.4	2.48	21.84	1626
Co(OH)_2_@PLDH-48	12.2	2.61	16.45	1353.2
