# Supplementary material for: Hierarchical Co-based Porous Layered Double Hydroxide Arrays Derived via Alkali Etching for High-performance Supercapacitors
Source: Sci Rep. 2015 Aug 17;5:13082. doi: 10.1038/srep13082 (PMC4538401; doi:10.1038/srep13082)
Supplement: Supplementary Information [file srep13082-s1.doc]

**Supporting information**

Hierarchical Co-based Porous Layered Double Hydroxide Arrays Derived via Alkali Etching for High-performance Supercapacitors

Nasser Abushrenta, Xiaochao Wu*, Junnan Wang, Junfeng Liu* & Xiaoming Sun

State Key Laboratory of Chemical Resource Engineering, Beijing University of Chemical Technology, Beijing 100029, China.

**Corresponding author. E-mail: wuxiaochao71622@gmail.com, ljf@mail.buct.edu.cn,*

*
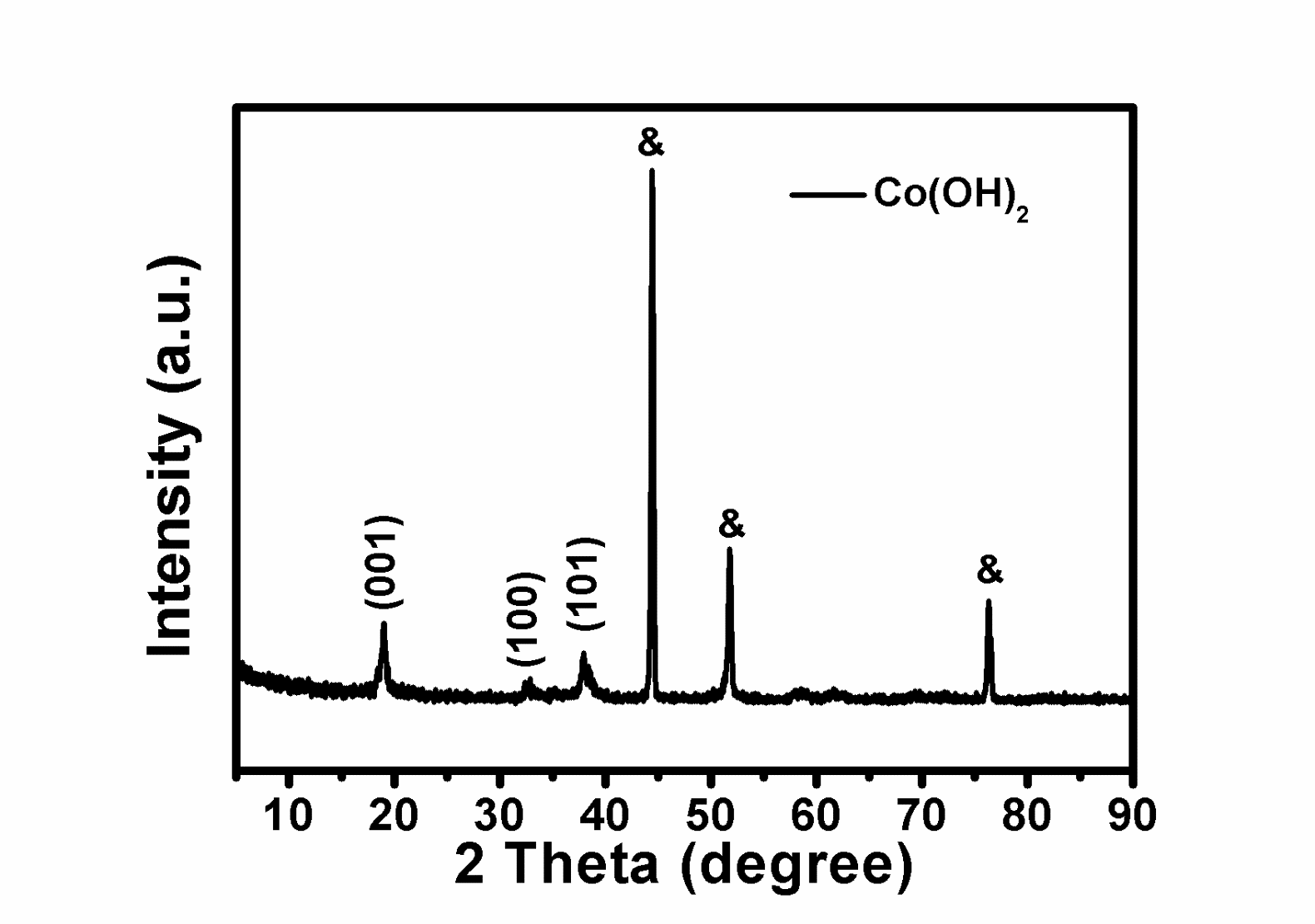
*

**Figure S1**. XRD pattern for the Co(OH)_2_ NS arrays


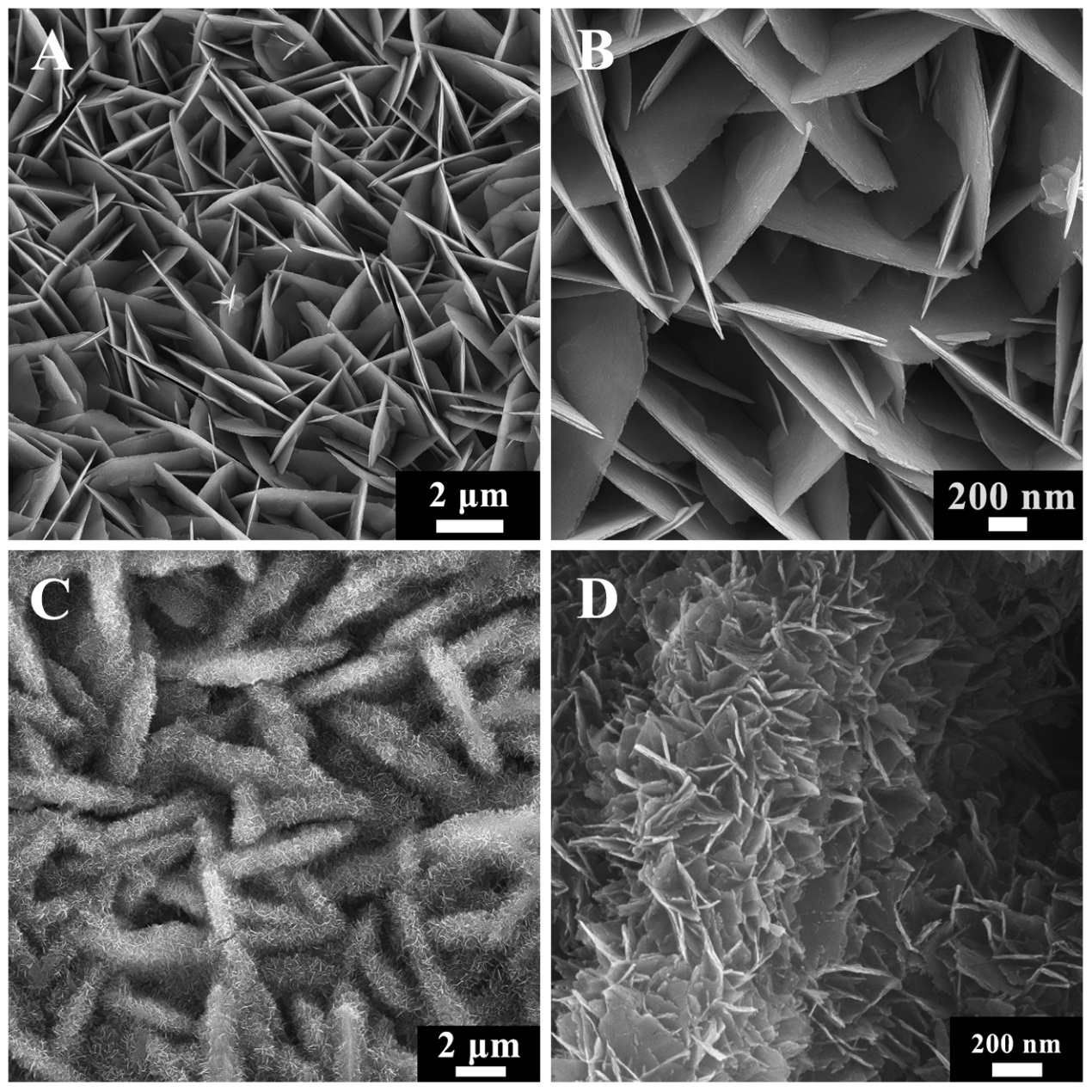


**Figure S2**. Low and high magnification SEM images of (A, B) the Co(OH)_2_ NS arrays, (C, D) Co(OH)_2_@CoAl LDH NS arrays.


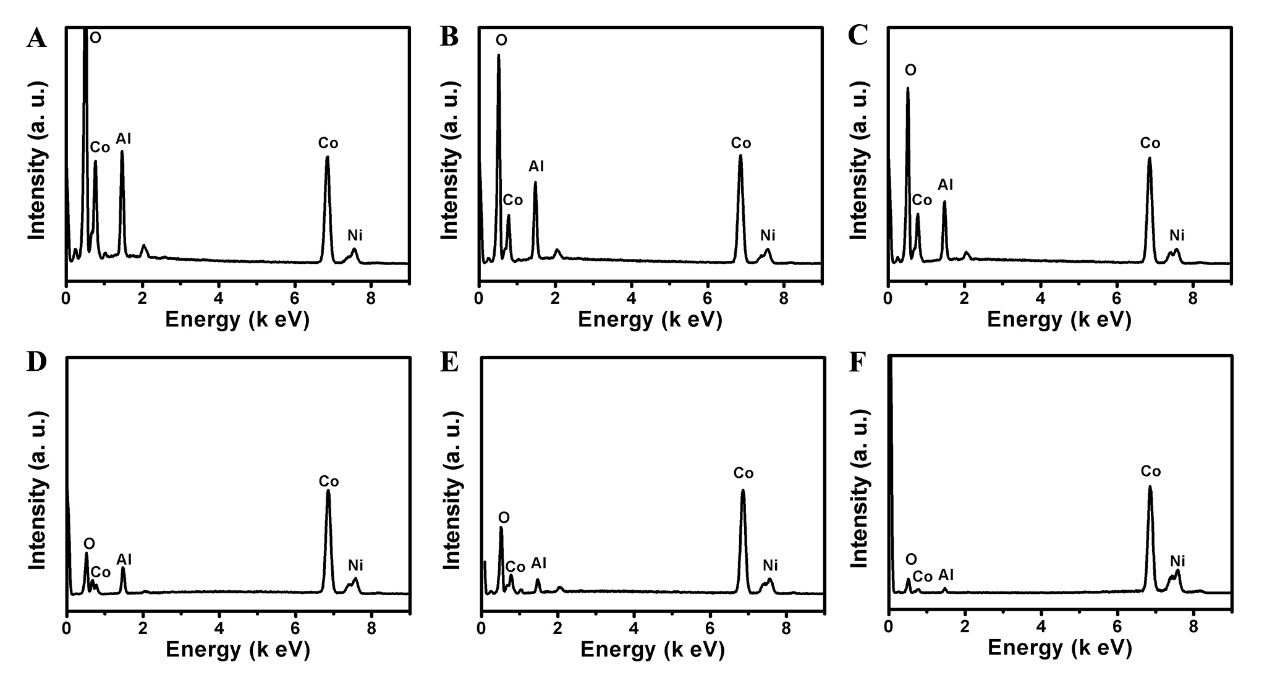


**Figure S3**. EDS of various samples with different alkali etching time, (A) Co(OH)_2_@CoAl LDH; (B) Co(OH)_2_@PLDH-6; (C) Co(OH)_2_@PLDH-12; (D) Co(OH)_2_@PLDH-18; (E) Co(OH)_2_@PLDH-24; (F) Co(OH)_2_@PLDH-48.

**Table S1**. Element content ratio of Co and Al in various samples with different alkali etching time.

| Alkali etching time  (hours) | 0 | 6 | 12 | 18 | 24 | 48 |
| --- | --- | --- | --- | --- | --- | --- |
| Element content  ratio of Co/Al | 0.98 | 1.3 | 1.6 | 2.83 | 5.75 | 18.44 |


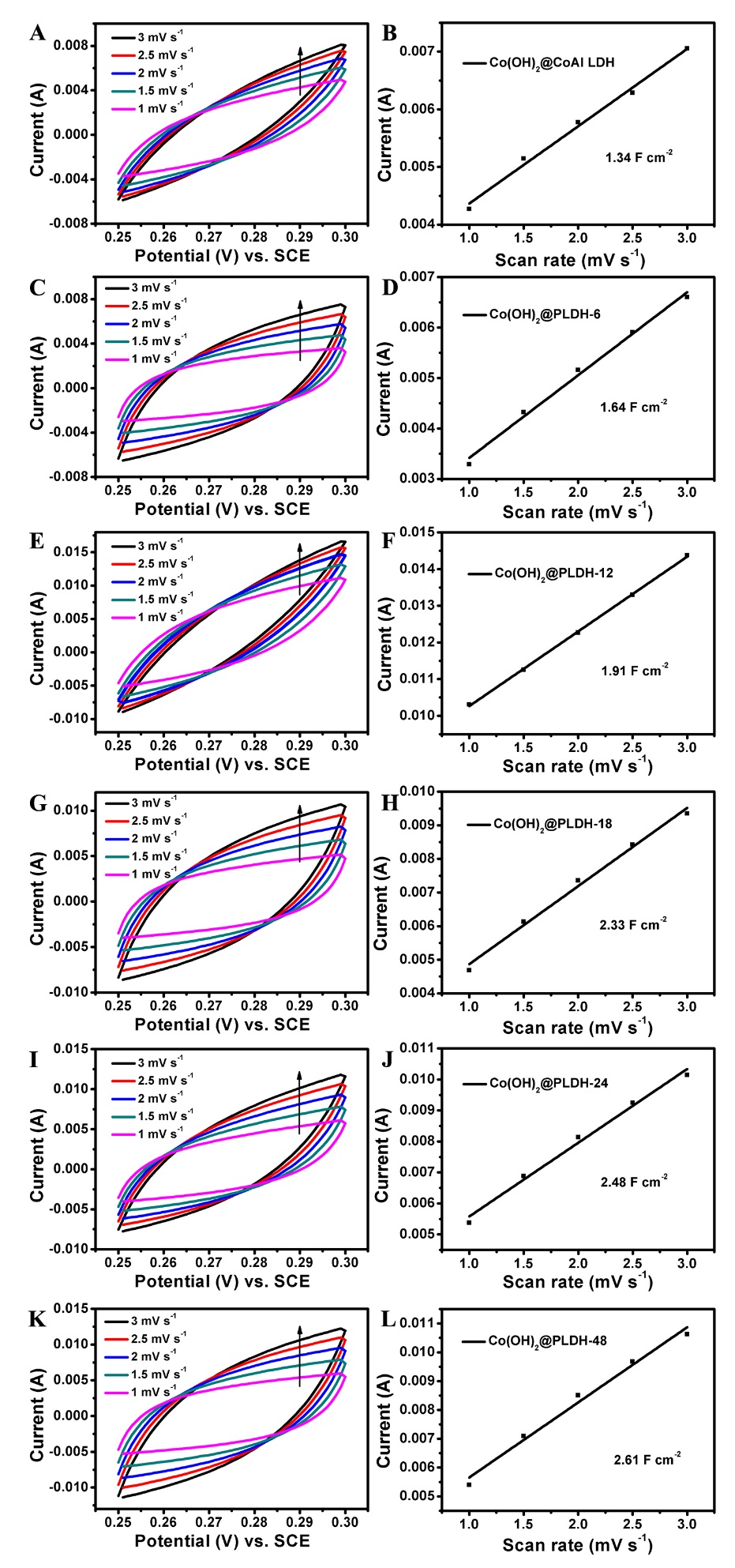


**Figure S4**. EDLC measurements of Co(OH)_2_@CoAl LDH and Co(OH)_2_@PLDH-X, and the corresponding current-scan rate curves.


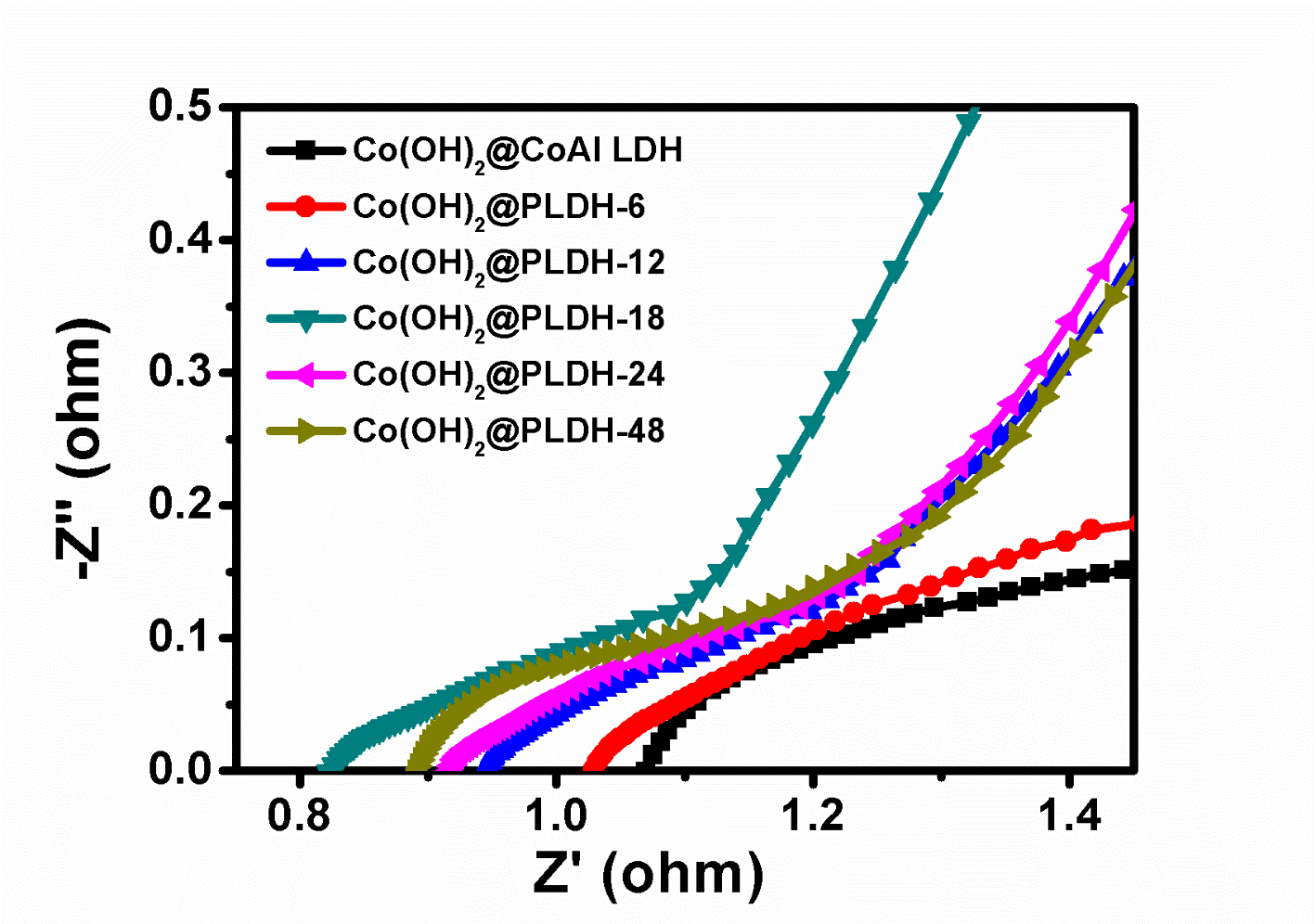


**Figure S5**. EIS plots in high frequency range


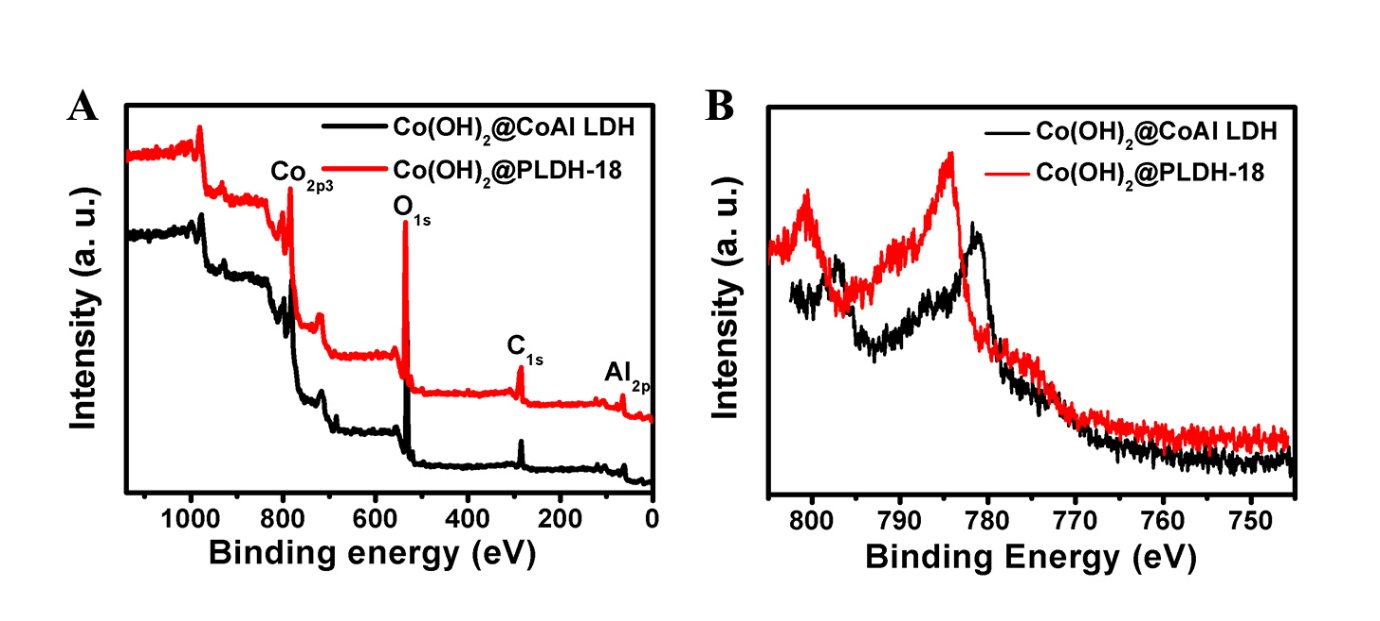


**Figure S6**. XPS pattern of the Co(OH)_2_@CoAl LDH and Co(OH)_2_@PLDH-18.


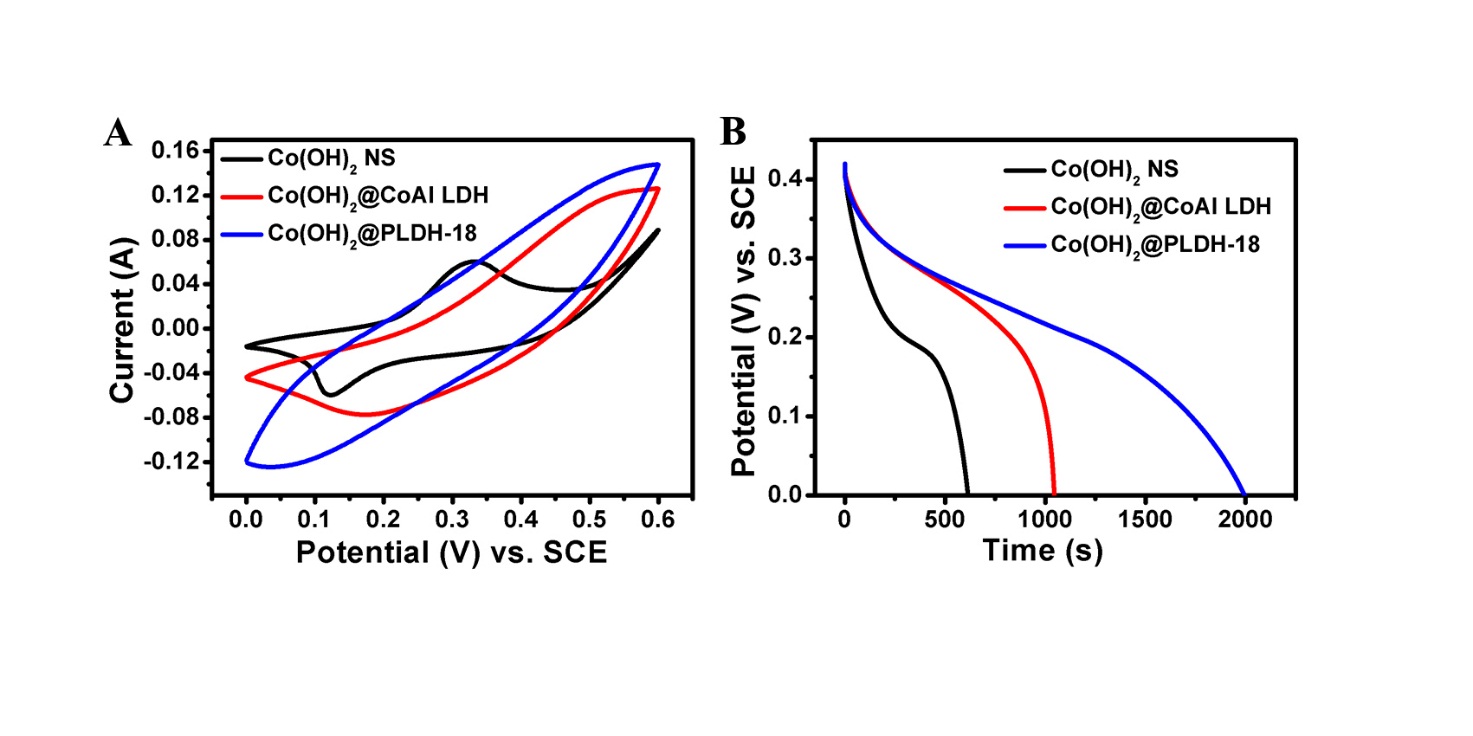


**Figure S7**. Electrochemical characterization of the Co(OH)_2_ NSs, Co(OH)_2_@CoAl LDH NSSAs and Co(OH)_2_@PLDH-18. (A) CV curves at 10 mV s^-1^; (B) galvanostatic discharge curves at 5mA cm^-2^.


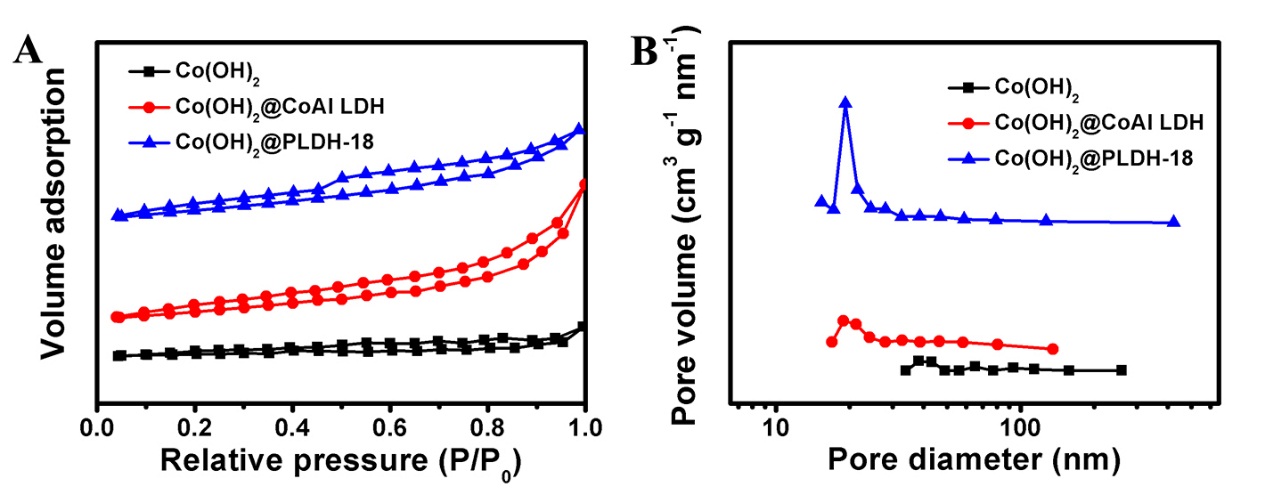


**Figure S8**. (A) The N_2_ adsorption/desorption isotherms; and (B) pore size distributions.

**Table S2**. The specific surface area of various samples.

| Samples | Specific surface area (m^2^ g^-1^) |
| --- | --- |
| Co(OH)_2_ | 3.5 |
| Co(OH)_2_@CoAl LDH | 15.5 |
| Co(OH)_2_@PLDH-18 | 25.2 |
